# Risk factors and management strategies in acute promyelocytic leukemia: a real-world single-center retrospective study

**DOI:** 10.3389/fmed.2026.1752909

**Published:** 2026-03-09

**Authors:** Yafeng Jiang, Haiying Zhong, Huihui Wang, Doudou Tang, Mingjie Chen, Hongling Peng, Guangsen Zhang, Yewei Wang

**Affiliations:** 1 Department of Hematology, The Second Xiangya Hospital, Central South University, Changsha, China; 2 Department of Respiratory Medicine, Hunan Province Chest Hospital, Changsha, China; 3 Shanghai NewCore Biotechnology Co., Ltd., Shanghai, China

**Keywords:** acute promyelocytic leukemia (APL), CD2 expression, early death, hyperleukocytosis, prognostic factors

## Abstract

**Background:**

Acute promyelocytic leukemia (APL) has become a highly curable malignancy with the advent of targeted therapies. However, early death (ED), predominantly caused by severe hemorrhagic complications, continues to be the most significant obstacle to achieving high survival rates in real-world clinical practice, where outcomes often lag behind those reported in controlled trials.

**Aims:**

This retrospective study aimed to identify the key clinical and laboratory factors associated with ED and overall survival (OS) in a cohort of newly diagnosed APL patients. The study also sought to evaluate the impact of current supportive care strategies and identify opportunities for improved management.

**Methods:**

This single-center, retrospective analysis included 132 patients newly diagnosed with APL between June 2017 and January 2023. Data on demographics, baseline laboratory values, immunophenotyping, treatment, and outcomes were collected. Univariate and multivariate logistic regression analyses were used to identify independent predictors of ED, while Kaplan-Meier and Cox regression models were used to assess survival outcomes.

**Results:**

The ED rate was 12.88% (17 patients), with hemorrhage being the primary cause. Multivariate analysis identified three independent risk factors for ED: a high white blood cell (WBC) count (>201 × 10^9^/L), prolonged prothrombin time (PT), and CD2 positivity. While severe thrombocytopenia was common, it was not an independent predictor, likely due to an aggressive platelet transfusion policy. Elevated lactate dehydrogenase (LDH) levels were significantly associated with poorer OS. Furthermore, all relapses in the high-risk group involved the central nervous system (CNS).

**Conclusions:**

This study found that high initial WBC count, prolonged PT, and CD2 positivity are critical independent predictors of ED in APL. The findings underscore the necessity of prompt diagnosis, risk-adapted therapy, and comprehensive multidisciplinary care, including aggressive management of coagulopathy. Enhanced CNS prophylaxis should be considered for high-risk patients to prevent relapse.

## Introduction

Acute promyelocytic leukemia (APL), a distinct subtype of acute myeloid leukemia (AML), is defined by the reciprocal translocation t(15;17)(q24.1;q21.2), resulting in the formation of the PML/RARα fusion gene (1, 2). This unique genetic alteration is not only central to the pathogenesis of APL but also confers remarkable sensitivity to differentiation therapy, particularly with all-trans retinoic acid (ATRA) and arsenic trioxide (ATO) (3, 4). Historically, APL was considered among the most fatal forms of leukemia, yet has been transformed into the most curable AML subtype through ATRA/ATO-based regimens (5), with recent clinical trials reporting >90% long-term survival (4, 6). Consequently, APL stands as a paradigm of targeted therapy success in hematologic malignancies (7).

However, this high curability is counterbalanced by a persistent and well-documented challenge: early death (ED) (8). The majority of EDs-typically defined as mortality within the first 30 days of diagnosis-are pre-dominantly attributed to hemorrhagic complications, particularly involving the central nervous system or lungs (9). Although ED rates are low in tightly controlled clinical trials, real-world data reveal a more concerning scenario, with ED rates ranging from 10 up to 40% in some cohorts (8, 10). Thus, ED remains the principal barrier to improving overall survival (OS) in APL, underscoring the urgent need for timely diagnosis and intervention to fully realize the curative potential of contemporary therapies.

Encouragingly, aggregated evidence indicates that the global ED rate in APL has decreased over recent years (11, 12), reflecting advancements in disease awareness, prompt diagnosis, and optimized supportive care strategies. Multicenter cohort studies (12), registry-based analyses (11), and international collaborations (13) all support this downward trend in many populations. Despite such progress, these improvements have not been universal. Notably, population-based studies from Sweden have reported persistently high ED rates, even in the modern era of APL therapy and across all age groups (14). This persistent risk accentuates the complexity of APL management and the necessity to explore the multifactorial causes behind early mortality, particularly among diverse patient populations with varying access to care and clinical resources.

A deeper exploration of contemporary risk factors for ED in APL is therefore warranted. Several variables have been consistently associated with increased ED risk, including older age at diagnosis, hyperleukocytosis (elevated white blood cell count), poor performance status, and the presence of severe coagulopathy at presentation (6, 10, 15). The relative impact of these factors may shift with evolving treatment protocols and improvements in transfusion support (16), especially as real-world patient populations and healthcare system capacities frequently diverge from the controlled environments of clinical trials. Understanding the interplay of these risk variables is essential for identifying patients at highest risk of early mortality, thereby enabling optimization of supportive care and therapeutic interventions.

In this context, we conducted a retrospective real-world study at our center, including all patients diagnosed with APL over a 6-year period. Our primary objective was to delineate clinical and laboratory factors associated with ED and to integrate our findings with the broader landscape of published literature. By comparing our institutional data with historic and multinational experiences, we aimed to elucidate ongoing drivers of ED in APL, assess the impact of interventions such as aggressive platelet transfusion, and highlight opportunities to improve early management. Through this work, we hope to contribute meaningful evidence toward optimizing APL care and closing the gap between curability and cure.

## Methods

### Study design and patient population

This was a single-center, retrospective cohort study that included 132 patients with newly diagnosed APL, admitted to the Second Xiangya Hospital of Central South University between June 2017 and January 2023. The diagnosis of APL was established based on bone marrow morphology, cytogenetic analysis, and molecular confirmation, in accordance with the 2016 World Health Organization (WHO) criteria. Inclusion criteria required: (1) newly diagnosed, previously untreated APL; (2) confirmed diagnosis by characteristic cytomorphology and demonstration of t(15;17)(q22;q21), and PML/RARα fusion by reverse transcription polymerase chain reaction (RT-PCR) or fluorescence *in situ* hybridization (FISH). Patients with incomplete clinical data or secondary APL were excluded.

Demographic data (age, sex), clinical presentations (including hemorrhagic manifestations and coagulopathy), and baseline laboratory parameters were collected from medical records at diagnosis. Baseline laboratory data included white blood cell (WBC) count, hemoglobin, platelet count, prothrombin time (PT), activated partial thromboplastin time (APTT), fibrinogen, fibrin degradation products (FDP), D-dimer, albumin, creatinine (Cr), lactate dehydrogenase (LDH), and C-reactive protein (CRP). Additional molecular and cytogenetic data—including immunophenotyping, PML/RARα status, and FLT3 mutation status—were documented. Informed consent was obtained, and the study protocol was approved by the institutional review board.

### Definitions

ED was defined as death from any cause within 30 days of initial admission. OS was calculated from the diagnosis date until death from any cause or the last follow-up. Event-free survival (EFS) was defined as the time from achievement of first complete remission to relapse, or death, or to last follow-up in patients without an event.

Risk stratification was applied using the Sanz score: low risk (WBC ≤ 10 × 10^9^/L, platelet >40 × 10^9^/L), intermediate risk (WBC ≤ 10 × 10^9^/L, platelet ≤ 40 × 10^9^/L), and high risk (WBC >10 × 10^9^/L). This classification guided the selection and intensity of therapeutic regimens.

### Treatment protocols and supportive care

All patients received induction therapy comprising ATRA (25 mg/m^2^/day, orally in two divided doses) initiated promptly upon suspicion of APL, combined with anthracycline-based chemotherapy (idarubicin or daunorubicin) and/or ATO (0.15 mg/kg/day) according to institutional protocols. Supportive management included aggressive correction of coagulopathy with transfusion of platelets, fresh frozen plasma, and cryoprecipitate, aiming for a platelet count >30 × 10^9^/L and fibrinogen >1.5 g/L, based on standardized ISTH DIC scoring. Prophylactic corticosteroids (prednisone 0.5 mg/kg/day) were administered from day 1 until the end of induction therapy to prevent differentiation syndrome.

Central nervous system (CNS) prophylaxis was provided for patients according to risk stratification. Measures to reduce treatment abandonment included social and financial support tailored to individual needs. After induction, consolidation and maintenance therapy were given in accordance with institutional protocols, guided by risk stratification and minimal residual disease (MRD) monitoring where available.

Patients were systematically monitored during treatment and follow-up for hematological and molecular remission status, adverse events, relapse, and survival. All hemorrhagic, infectious, and thrombotic events were documented prospectively.

### Statistical analysis

Clinical and laboratory variables were analyzed to identify factors associated with ED and survival outcomes. Relationships between baseline variables and ED were assessed by univariate and multivariate logistic regression; variables with *p* < 0.05 in univariate analysis were included in multivariate models to control for confounding factors. Survival curves were generated using the Kaplan-Meier method and compared using the log-rank test. Cox proportional hazards regression was performed to evaluate the association between baseline variables and OS. Statistical significance was set at *p* < 0.05. Analyses were performed with SPSS software (version 27.0; IBM Corp., Armonk, NY, USA).

## Results

### Baseline characteristics

A total of 132 newly diagnosed APL patients admitted to the Second Xiangya Hospital of Central South University between June 2017 and January 2023 were enrolled in this study. The median age was 41 years (range: 14–70), with 38 patients aged ≥50 and 10 patients aged ≥60. There were 76 males and 56 females (Table 1). Sanz risk stratification classified 35 patients as high-risk, 73 as intermediate-risk, and 24 as low-risk.

**Table 1:** Baseline patient characteristics.

**Characteristic**	***n***	**Median (range)**	***n* (%)**
*N*			132 (100%)
Age, *y*	132		
≥50			38 (28.8%)
<50			94 (71.2%)
Gender	132		
Male			76 (57.6%)
Female			56 (42.4%)
WBC count, × 10^9^/L	132		
≤5			75 (56.8%)
>5–10			22 (16.7%)
>10–20			14 (10.6%)
>20–50			11 (8.33%)
>50			10 (7.57%)
Hemoglobin, g/L	132		
≤100			109 (82.6%)
>100			23 (17.4%)
Platelet count, × 10^9^/L	132		
≤40			103 (78.0%)
>40			29 (22.0%)
Sanz risk	132		
High			35 (26.5%)
Intermediate			73 (55.3%)
Low			24 (18.2%)
BM leukemia cells (%)	127	86.5 (23.7–99)	
pB blasts (%)	124	60 (0–97)	
Bleeding	132		
No			24 (18.2%)
Mild			81 (61.4%)
Moderate			15 (11.4%)
Severe			12 (9.09%)
Fever	132		
Yes			63 (47.7%)
No			69 (52.3%)
PML/RARα isoform	126		
L/S isoform			114 (90.5%)
Variant isoform			12 (9.5%)
FLT3 status	91		
FLT3-ITD			22 (24.2%)
FLT3-TKD			7 (7.69%)
wild type			62 (68.1%)
Induction treatment	129		
ATRA			6 (4.65%)
ATRA + ATO			12 (9.3%)
ATRA + IDA/DNR			87 (67.4%)
ATRA + ATO + IDA/DNR			24 (18.6%)

At diagnosis, 108 patients exhibited bleeding manifestations, including mucocutaneous hemorrhage (*n* = 106), hemoptysis (*n* = 6), melena (*n* = 4), hematuria (*n* = 3), intracranial hemorrhage (*n* = 6), menorrhagia (*n* = 7), and bleeding at other sites (*n* = 5). Fever was present in 63 patients at diagnosis.

All 132 patients were confirmed to be PML/RARα positive. Among them, 126 patients were analyzed by RT-PCR, revealing the L/S subtype in 114 cases (90.5%) and variant subtypes in 12 cases (9.5%). FISH analysis was performed in 37 patients, of whom 36 were positive for PML/RARα.

The FLT3 mutation status was determined for 91 patients, identifying FLT3-ITD mutations in 22 patients and FLT3-TKD mutations in seven patients.

Three patients died before the initiation of ATRA therapy. Two of them were initially admitted to the neurology department because of acute hemorrhagic events and died shortly thereafter before APL was recognized. The third patient was admitted to the hematology department for pancytopenia and died before diagnosis of APL. The remaining 129 patients received treatment. Among them, six patients received only ATRA; all of these six patients died from hemorrhage before receiving ATO or chemotherapy. Twelve patients (9.3%) were treated with ATRA combined with ATO, 87 patients (67.4%) received ATRA combined with idarubicin- or daunorubicin-based chemotherapy, and 24 patients (18.6%) were treated with a combination of ATRA, ATO, and idarubicin- or daunorubicin-based chemotherapy.

### Early death

Seventeen patients experienced ED, corresponding to a mortality rate of 12.88%. The median age of ED patients was 46 years (range: 17–68). ED rate was 11.7% (11/94) in patients < 50 years, 15.8% (6/38) in patients ≥50 years, and 30% (3/10) in those >60 years.

The ED rate was 28.6% (10/35) in the high-risk group, 6.85% (5/73) in the intermediate-risk group, and 8.33% (2/24) in the low-risk group. The mortality rate was obviously increased in the high-risk group (Table 2).

**Table 2:** Baseline characteristics of early death and non-early death patients.

**Factor**	**Early death patients**	**All other patients**
*N*	17 (12.88%)	115 (87.12%)
Age, median	46 (17–68)	40 (14–70)
Age >60 years, *n*	3 (17.6%)	7 (6.09%)
Female, *n*	8 (47.1%)	48 (41.7%)
Sanz risk		
High	10 (58.8%)	25 (21.7%)
Intermediate	5 (29.4%)	68 (59.1%)
Low	2 (11.8%)	22 (19.1%)
WBC × 10^9^/L, median	16.16	2.64
Platelet × 10^9^/L, median	21	21
Hemoglobin (g/L), median	75	73
PT prolongation >3s, *n*	12 (70.6%)	38 (33%)
PT prolongation >6s, *n*	5 (29.4%)	11 (9.56%)
Fib < 1.0g/L, *n*	3 (17.6%)	23 (20%)
D-dimer ratio >10, *n*	12 (80%)	74 (67.3%)
FDP ratio >10, *n*	10 (62.5%)	55 (49.1%)
DIC score ≥5, *n*	15 (93.8%)	77 (68.1%)
DIC score ≥6, *n*	9 (56.2%)	39 (34.5%)
DIC score ≥7, *n*	5 (31.2%)	16 (14.1%)
LDH (U/L), median	605 (249–1,724)	280 (131–1,147)
BM leukemia cells, median	89%	86.50%
pB blasts, median	67%	56%
CD2 expression	4 (36.4%)	8 (7.55%)
CD56 expression	1 (9.09%)	12 (11.3%)

The primary cause of ED was hemorrhage (*n* = 15), including intracranial hemorrhage in nine patients (52.9%), alveolar hemorrhage in six patients (35.3%), and concurrent intracranial and alveolar hemorrhage in two patients. Other causes included cerebral infarction (*n* = 1, 5.88%) and pulmonary infection with respiratory failure (*n* = 1, 5.88%).

Three patients died within 3 days of admission before receiving ATRA treatment. Two of these patients were initially admitted to the neurology department for impaired consciousness, were diagnosed with intracranial hemorrhage, with APL being the final diagnosis. Eleven deaths occurred within 7 days (intracranial hemorrhage *n* = 4, alveolar hemorrhage *n* = 4, combined intracranial and alveolar hemorrhage *n* = 1, cerebral infarction *n* = 1, and pulmonary infection with respiratory failure *n* = 1). Two additional deaths occurred within 14 days, caused by alveolar hemorrhage (*n* = 1) and combined intracranial and alveolar hemorrhage (*n* = 1).

Among 35 patients with WBC >101 × 10^9^/L, 10 died; five of 10 patients with WBC >501 × 10^9^/L died, demonstrating increasing ED rates with higher leukocytosis in high-risk patients (Table 3).

**Table 3:** The association between WBC counts at diagnosis and early death.

**WBC counts at diagnosis**	**No**	**Early death (%)**
10~ < 20	14	2 (14.3%)
20~ < 50	11	3 (27.3%)
≥50	10	5 (50%)

Among 61 patients with platelets < 201 × 10^9^/L, eight experienced ED; for the 25 patients with platelets < 101 × 10^9^/L, four died early. Hypofibrinogenemia (< 1.5 g/L) was present in 55 patients (ED = 10), including 26 with levels < 1.0 g/L (ED = 3).

Immunophenotyping was performed on 117 patients. Among the 13 CD56-positive patients, one died early (7.69%), whereas among the 104 CD56-negative patients, 16 died early (15.4%). Among the 12 CD2-positive patients, four died early (33.3%), compared to 13 EDs among the 105 CD2-negative patients (12.4%).

As shown in Table 4A, univariate logistic regression analysis identified the following factors as being significantly associated with ED: WBC count >201 × 10^9^/L (OR 6.974, *p* = 0.0006), prothrombin time (PT) prolongation >6s (OR 4.298, *p* = 0.0198), creatinine (Cr) >70 μmol/L (OR 4.5, *p* = 0.0134), lactate dehydrogenase (LDH) >500 U/L (OR 11.33, *p* = 0.0003), and CD2 positivity (OR 7, *p* = 0.0074). Multivariate logistic regression analysis (Table 4B) revealed that WBC count >201 × 10^9^/L (OR 10.11, *p* = 0.0236), PT prolongation >6s (OR 17.15, *p* = 0.0137), and CD2 positivity (OR 18.5, *p* = 0.0262) were independent prognostic factors for ED in patients with APL. Cr >70 μmol/L and LDH >500 U/L were excluded from the final model.

**Table 4:** Univariate and multivariate analysis for the one-month mortality.

**Parameters**	**No**	**OR**	**95% CI**	***p*-value**
**(A) Univariate analysis**				
WBC count >201 × 10^9^/L	21	6.974	2.270–21.61	0.0006
Platelet count < 40 × 10^9^/L	103	0.633	0.2117–2.145	0.4301
Platelet count < 10 × 10^9^/L	25	1.377	0.3604–4.355	0.606
DIC score ≥7	21	2.756	0.7849–8.722	0.0928
PT prolongation >6s	16	4.298	1.183–14.37	0.0198
FDP ratio >5	96	5.122	0.9641–94.76	0.1222
D-dimer ratio >10	71	3.547	0.9273–23.32	0.1053
Fib < 1.0g/L	26	0.9231	0.1998–3.161	0.9065
Albumin < 35 g/L	21	2.813	0.8013–8.898	0.0863
Cr >70 μmol/L	58	4.5	1.467–16.88	0.0134
LDH >500 U/L	18	11.33	3.119–44.60	0.0003
BM leukemia cells >90%	45	2.739	0.8894–8.871	0.0803
pB blasts >80%	40	3.352	0.9998–12.05	0.0515
CD56 positive	13	0.7833	0.04090–4.666	0.8232
CD2 positive	12	7	1.572–28.98	0.0074
**(B) Multivariate analysis**				
WBC count >201 × 10^9^/L		10.11	1.463–98.46	0.0236
PT prolongation >6s		17.15	2.008–229.6	0.0137
LDH >500 U/L		2.232	0.2818–16.07	0.4256
CD2 positive		18.5	1.549–341.4	0.0262
Cr >70 μmol/L		4.295	0.5922–50.33	0.1783

### Overall survival

**Figure 1:**
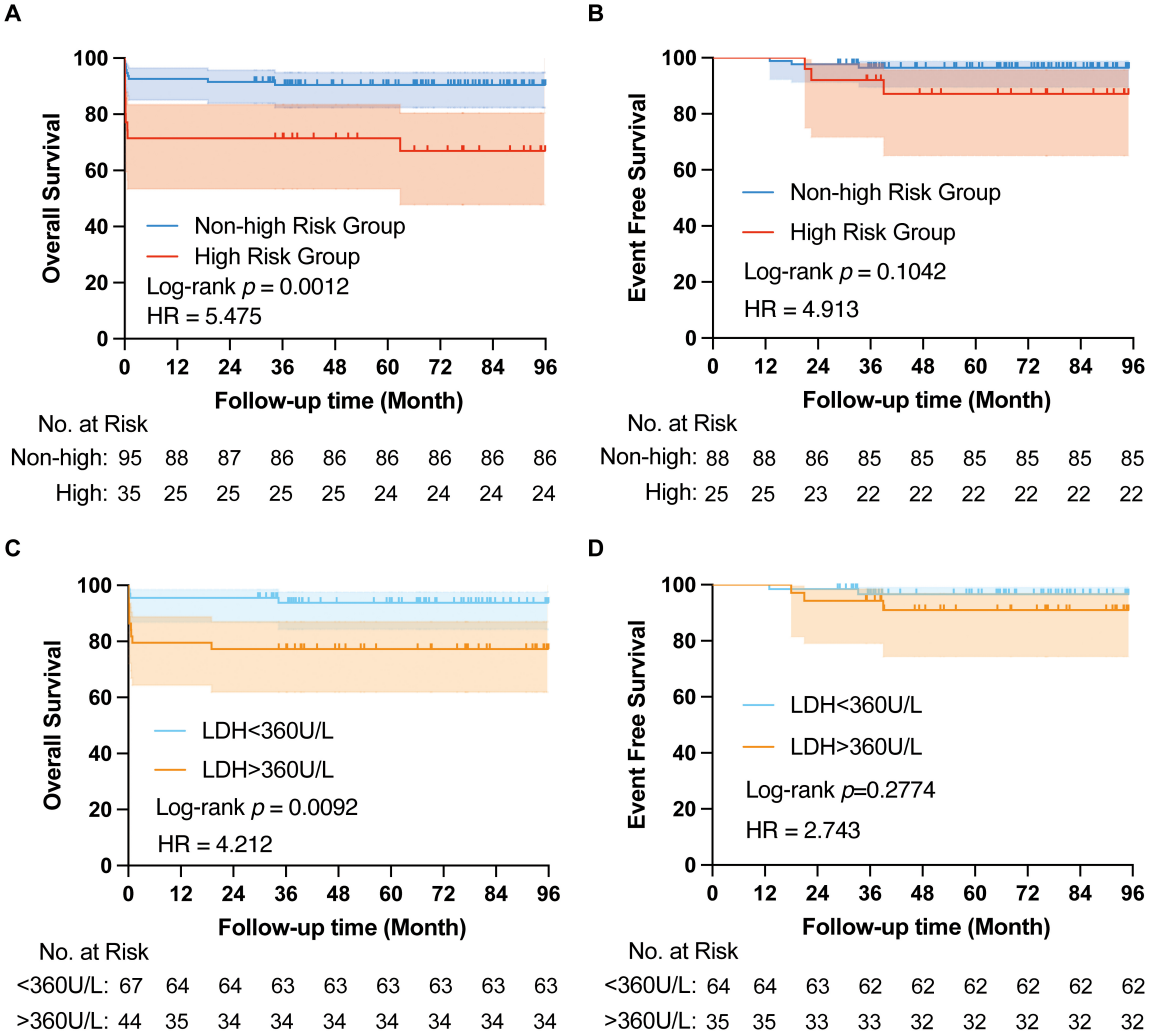
Survival situation based on prognostic status and LDH levels. Overall survival **(A)** and Event free survival **(B)** based on high risk and non-high-risk group. Overall survival **(C)** and Event free survival **(D)** based on LDH levels.

The median follow-up duration was 67 months for all patients and 70 months for non-ED patients. For the entire cohort, the 1-year, 3-year, and 5-year OS rates were 86.2 ± 3%, 85.3 ± 3.1%, and 84.1 ± 3.3%, respectively. For patients who survived the induction period, the 1-year and 3-year EFS rates were 99.1 ± 0.9% and 94.4 ± 2.2%, respectively. The 3-year OS rates for the high-risk vs. non-high-risk groups were 71.4%±7.6% and 90.4%±3%, respectively (Figure 1A), while the 3-year EFS rates were 87.2 ± 7.0% and 96.5 ± 2.0%, respectively (Figure 1B).

Of the 115 non-ED patients, two were lost to follow-up, and five relapsed at a median of 21 months (range: 13–39 months). Two of the relapsed patients died (40%), and three are still alive. Among the relapsed patients, three were from the high-risk group (two with CNS relapse and one with simultaneous bone marrow and CNS relapse), and two were from the intermediate-risk group (both with bone marrow relapse). Two patients transformed to another type of acute leukemia; one was diagnosed with AML M4 subtype 33 months after the APL diagnosis (this patient died of severe infection following induction chemotherapy), and another developed AML M2 subtype 42 months after the APL diagnosis (this patient was discharged after bone marrow recovery following chemotherapy and was subsequently lost to follow-up).

A Cox proportional hazards model showed that LDH level greater than 360 U/L was associated with a poorer OS (Table 5; Figures 1C, D).

**Table 5:** Univariate Cox proportional hazard model for overall mortality.

**Parameters**	**HR**	**95%CI**	***p* value**
Gender	0.53	0.08–3.524	0.512
WBC counts	1.002	0.982–1.022	0.863
PT	1.287	0.84–1.972	0.247
DIC score	0.683	0.258–1.808	0.443
D-Dimer	1.031	0.979–1.087	0.247
LDH	1.004	1–1.007	0.043
pB blasts	1.003	0.97–1.038	0.842
BM leukemia cells	1.015	0.919–1.12	0.771
CD2 positive	2.011	0.252–16.018	0.509
Cr	1.022	0.991–1.053	0.169

## Discussion

**Figure 2:**
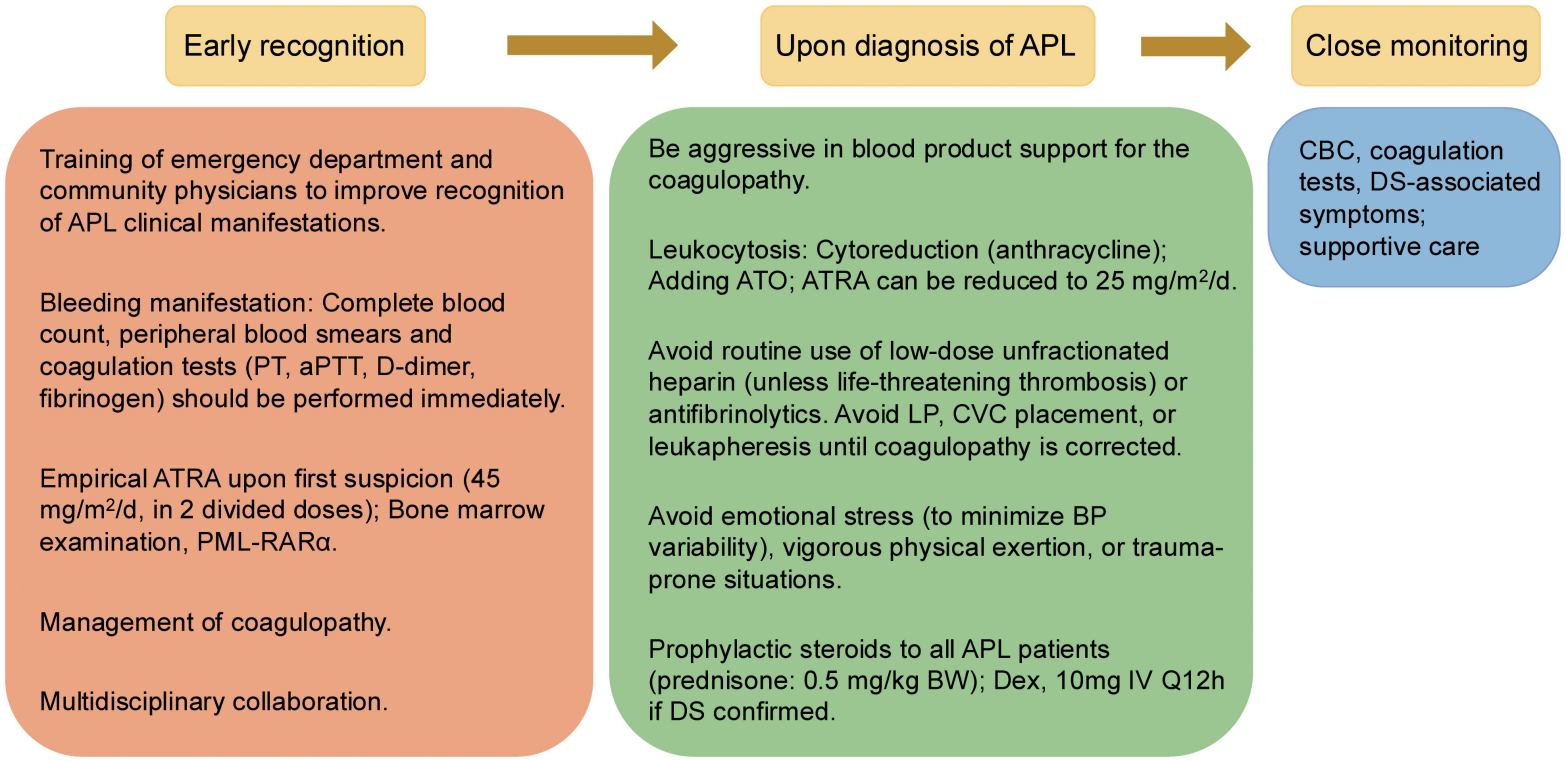
Proposed management of acute promyelocytic leukemia at diagnosis.

APL is uniquely associated with a substantial risk of early mortality, largely attributable to severe coagulopathy and life-threatening hemorrhagic events (17). Timely recognition and diagnosis are imperative, as delayed intervention significantly increases ED rates (18). Accordingly, improving the training of primary care professionals and emergency physicians is critical to facilitate prompt APL identification. While bleeding is a frequent initial manifestation in APL, the severity of hemorrhage often exceeds what would be expected from thrombocytopenia alone, reflecting the distinctive coagulopathic profile of the disease (19). Therefore, for any patient presenting with unexplained bleeding, comprehensive evaluation including complete blood count, coagulation studies, and peripheral blood smear should be promptly performed. If APL is suspected, empirical initiation of ATRA is essential, as delays increase the risk of ED (20). Multidisciplinary management, involving hematology consultation, remains crucial for optimal outcomes (see Figure 2). This retrospective analysis from our center provides new insights into prognostic markers and contemporary management of APL. The following discussion contextualizes our findings within the published literature, focusing on clinically relevant implications.

### Elevated WBC count as a predictor of early mortality

Our data confirm that a high presenting WBC count remains among the most robust and reliable independent predictors of ED in APL, concordant with previous studies (10, 14, 15, 21–23). Hyperleukocytosis heightens the risk of fatal hemorrhage, necessitating urgent initiation of differentiation therapy and, when indicated, cytoreductive chemotherapy. Major guidelines (e.g., NCCN Clinical Practice Guidelines in Oncology: Acute Myeloid Leukemia, Version 2.2025) and previous consensus (24, 25) recommend ATO in combination with induction therapy for hyperleukocytic patients. Close monitoring for differentiation syndrome (DS) and tumor lysis syndrome is warranted, with attention to fluid balance, electrolytes, and organ function. In alignment with clinical trial evidence (4, 26) and expert consensus (27), our practice included routine prophylactic prednisone for all ATRA-treated individuals, and notably, none of our EDs were related to DS. Our results reinforce the international standard of risk-adapted therapy and aggressive supportive care for patients with hyperleukocytosis (24, 28).

### Renal function: lower thresholds and outcome implications

Prior research has identified elevated serum creatinine as an adverse prognostic indicator in APL (14, 21). Although international criteria define renal dysfunction at creatinine >133 μmol/L (1.5 mg/ds), our study and others (23) indicate that even modest elevations are associated with poorer prognosis. Specifically, creatinine >133 μmol/L correlates with markedly increased 7-day mortality (29). Possible explanations include regional variation in baseline renal function and heightened susceptibility to early renal injury in APL. Even mild renal impairment can exacerbate DIC and elevate the risk of adverse effects from ATRA or ATO, underscoring the need for careful monitoring and individualized supportive care at the earliest sign of renal compromise.

### CD2 positivity identifies an adverse APL subset

A significant observation in our cohort is the independent adverse prognostic relevance of CD2 expression on APL blasts. This aligns with previous evidence linking CD2 positivity to microgranular variant (M3v) morphology, short-form PML/RARα transcripts, and higher leukocyte counts (30, 31). CD2+ APL is characterized by higher early mortality, lower complete remission rates, and increased relapse risk compared to CD2- cases (31–33). Mechanistic studies suggest that CD2+ APL arises from a more primitive hematopoietic progenitor and may involve altered immune microenvironment interactions (34). Recent work has implicated TIM-3+CD34+CD38– cells, expressing T-cell antigens TIM-3, as leukemia stem cells in APL with short-form PML/RARα transcripts, further associating this phenotype with ED risk (18, 35). The independent prognostic value of CD2 expression is based only on 12 CD2 positive APL cases. Further clinical studies with larger sample sizes are needed for confirmation.

### Prophylactic platelet transfusion as a protective factor

Although profound thrombocytopenia (< 101 × 10^9^/L) did not independently predict early mortality in our analysis, it remains associated with fatal hemorrhage in earlier studies (14, 15, 21, 23, 36). This discrepancy likely reflects the effectiveness of our center's aggressive platelet transfusion strategy, which reduces bleeding risk and may lessen the statistical impact of baseline platelet count. Recent data likewise question the independent prognostic role of platelet count in modern clinical practice (10). Our findings underscore the importance of maintaining platelet counts above guideline-recommended thresholds and suggest that ready access to transfusion support can shift mortality drivers in APL. This approach should be widely adopted in resource-sufficient settings to minimize hemorrhagic deaths.

### Fibrinogen, coagulopathy, and early death: beyond cryoprecipitate

While hypofibrinogenemia has been associated with ED (37), it was not independently predictive in our multivariate analysis, possibly owing to effective administration of fibrinogen concentrate and/or cryoprecipitate. In contrast, prolonged prothrombin time (PT) was a strong adverse prognostic marker, which was consistent with previous reports (29, 38). This highlights the need for timely correction not only of fibrinogen levels but also broader coagulation defects, potentially employing fresh frozen plasma or prothrombin complex concentrates (24, 39). Given the multifactorial etiology of APL-related DIC, further research should prioritize a comprehensive approach to coagulation management, in alignment with contemporary guidelines (24, 40, 41).

### LDH as a prognostic biomarker

Elevated lactate dehydrogenase (LDH) is consistently associated with increased hemorrhage risk (42, 43) and early mortality (21, 23, 44). In our cohort, LDH >360 U/L was linked to poorer OS, perhaps reflecting higher leukemic burden, subclinical tissue damage, or hypoxia related to DIC. This finding supports incorporating LDH assessment into early APL risk stratification to inform the need for intensified therapy, as corroborated by population-based studies (45).

### CNS relapse in high-risk APL: Challenges and prophylaxis

All relapses observed in our high-risk group involved CNS leukemia (CNSL; incidence: 12% [3/25]), which is rare overall but disproportionately affects high-risk cohorts. Reported CNSL rates without intrathecal (IT) prophylaxis range from 0% in low-risk to 5.5% in high-risk groups (46). Despite following current guidelines for CNS prophylaxis (e.g., IT methotrexate/cytarabine) (24, 25), three patients relapsed in the CNS after 2–4 prophylactic doses. Given that Chinese guidelines recommend 2–6 IT prophylaxis doses for high-risk APL (25), our findings suggest that more intensive regimens may be justified for these populations, supporting expert consensus for CNS prophylaxis in hyperleukocytic disease.

In this retrospective study, the cut-off values for continuous variables (e.g., WBC >201 × 10^9^/L, PT prolongation >6 s, LDH >360 U/L) were primarily determined based on established clinical standards and widely used prognostic thresholds in prior APL literature and guidelines (e.g., Sanz risk stratification, NCCN guidelines), which facilitate direct clinical applicability and comparison with existing studies.

Literature review of large-scale studies (*n* > 100; Table 6) reveals that although ED rates have declined over time (12), they remain in the range of 10–20% in most reports. Some studies from China report rates below 10%, which may exclude patients initially managed outside hematology, such as those with intracranial hemorrhage admitted to neurology; notably, two of our 17 EDs occurred in neurology wards.

**Table 6:** Literature (cases >100) regarding early death in adult APL.

**References**	**Type**	**Years**	**Total patients**	**ED rates %**	**OS%**	**High risk %**
NIS, US (47)	Database	2016–2019	3,800	12.10%	NA	NA
NCDB, US (48)	Database	2004–2015	7,190	12%	1-year OS:81%; 3-year OS:75%;	NA
SEER, US (49)	Database	1992–2007	1,400	17.30%	1-year OS:70.7%; 3-year OS:65.7%;	NA
SEER, US (11)	Database	2000–2014	2,962	17% (2010–2014)	4-year OS:73.4% (2010–2014)	NA
California, US (12)	Database	1999–2014	963	Total 27.2%; 22.74% (2011–2014)	NA	NA
US (20)	Multi-center	1992–2009	204	11%	NA	25%
Sweden (14)	Multi-center	1997–2013	195	26% (2009–2013)	NA	31% (2009–2013)
Sweden (15)	Multi-center	1997–2020	301	19.60%	NA	28%
Portugal (15)	Single center	2005–2019	129	18.60%	NA	28.7%
France (50)	Multi-center	2006–2011	399	9.6%	NA	27%
Brazil (51)	Multi-center	2003–2006	134	32%	55.3%	25%
Brazil (10)	Single center	2007–2018	112	22.30%	NA	37.5%
Latin America (13)	Multi-center	2006–2010	183	15%	2-year OS:80%	32%
Latin America (18)	Multi-center	2005–2020	806	14.6%	4-year OS:81%	36.7%
Hong Kong, China (45)	Multi-center	2007–2020	358	15.6%	5-year OS:68.6%	36%
Zhejiang, China (22)	Multi-center	2015–2019	1,233	8.20%	3-year OS:87.9%	23.4%
Suzhou, China (23)	Single center	2005–2019	570	7.54%	NA	27.4%
Henan, China (32)	Single center	2010–2019	219	5%	NA	35.6%
The present study	Single center	2018–2023	132	12.88%	5-year OS:84.1%	26.5%

This study has several limitations. First, it was conducted at a single center, which may limit the generalizability of the findings to other institutions or patient populations. Second, the relatively small number of early death cases within the cohort may have reduced the statistical power to detect certain associations. Third, the retrospective design carries inherent risks of selection bias and incomplete data collection. Finally, molecular data were not available for all patients, which may have limited the ability to fully assess the prognostic impact of genetic subtypes and mutations.

## Conclusion

In summary, this real-world analysis affirms established risk factors for early and overall mortality in APL, while highlighting the value of aggressive transfusion and comprehensive coagulation support in mitigating certain traditional prognostic markers. Persistently elevated PT, high initial WBC, CD2 positivity, high LDH, and the risk of CNS relapse in high-risk patients remain critical considerations in contemporary APL management. Our findings emphasize the importance of individualized, risk-adapted, and multidisciplinary approaches to optimize outcomes in APL.

## Data Availability

The original contributions presented in the study are included in the article/supplementary material, further inquiries can be directed to the corresponding author.
